# Supplementary material for: CryoROLE: describing large inter-domain rotation in single particle cryo-EM
Source: bioRxiv. 2026 Jul 4:2026.07.04.736454. Preprint. [Version 1] doi: 10.64898/2026.07.04.736454 (PMC13345010; doi:10.64898/2026.07.04.736454)
Supplement: 1 [file NIHPP2026.07.04.736454v1-supplement-1.pdf]

bioRxiv preprint doi: https://doi.org/10.64898/2026.07.04.736454; this version posted July 4, 2026. The copyright holder for this preprint (which was not certified by peer review) is the author/funder, who has granted bioRxiv a license to display the preprint in perpetuity. It is made available under a CC-BY 4.0 International license.

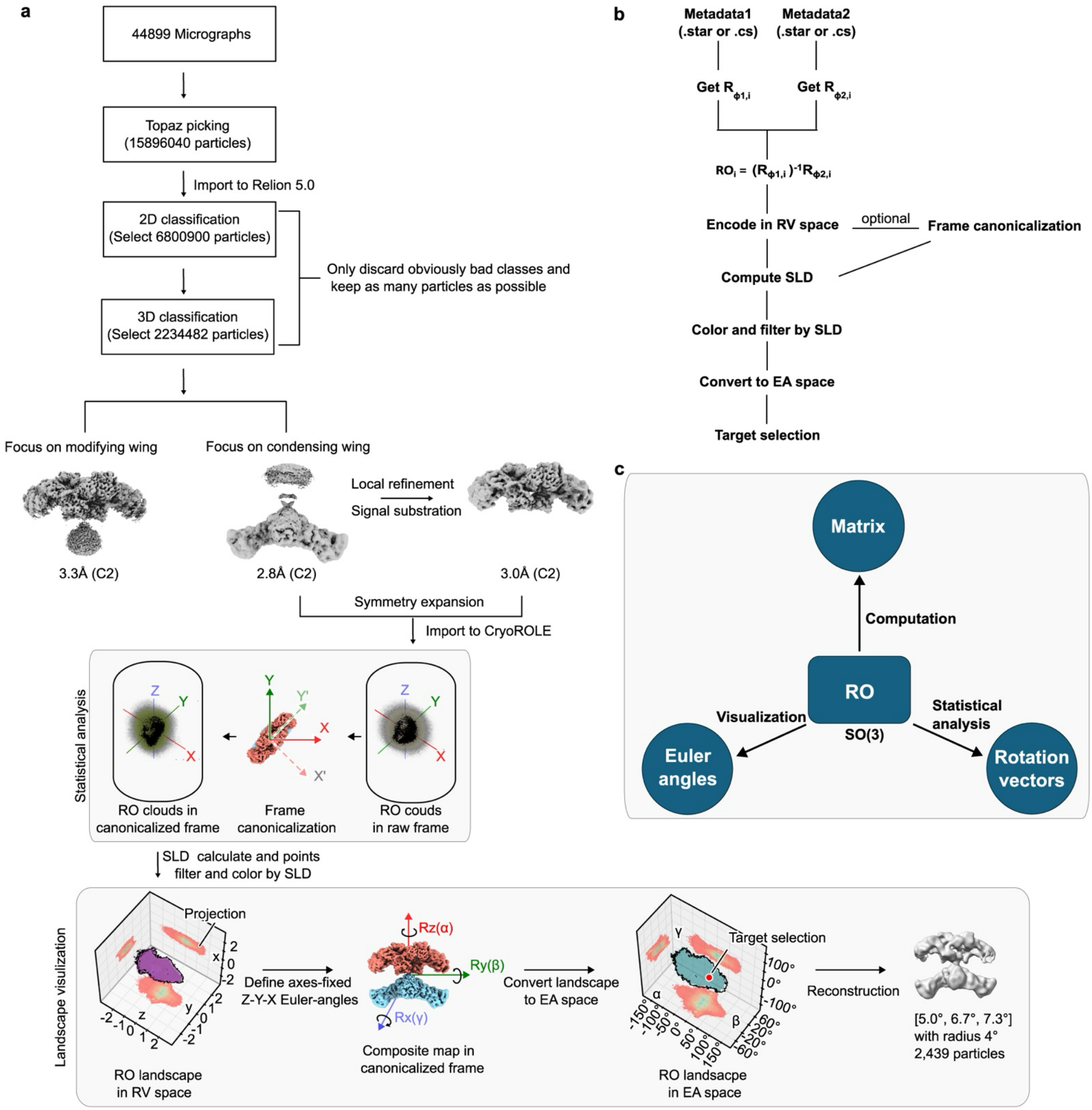

Supplementary Figure 1 Human FASN processing and cryoROLE workflow.
a, Workflow of processing FASN dataset and generation of RO landscape by cryoROLE. **b,** cryoROLE computational pipeline. **c,** RO representations used for computation, statistical analysis and visualization.

bioRxiv preprint doi: https://doi.org/10.64898/2026.07.04.736454; this version posted July 4, 2026. The copyright holder for this preprint (which was not certified by peer review) is the author/funder, who has granted bioRxiv a license to display the preprint in perpetuity. It is made available under a CC-BY 4.0 International license.

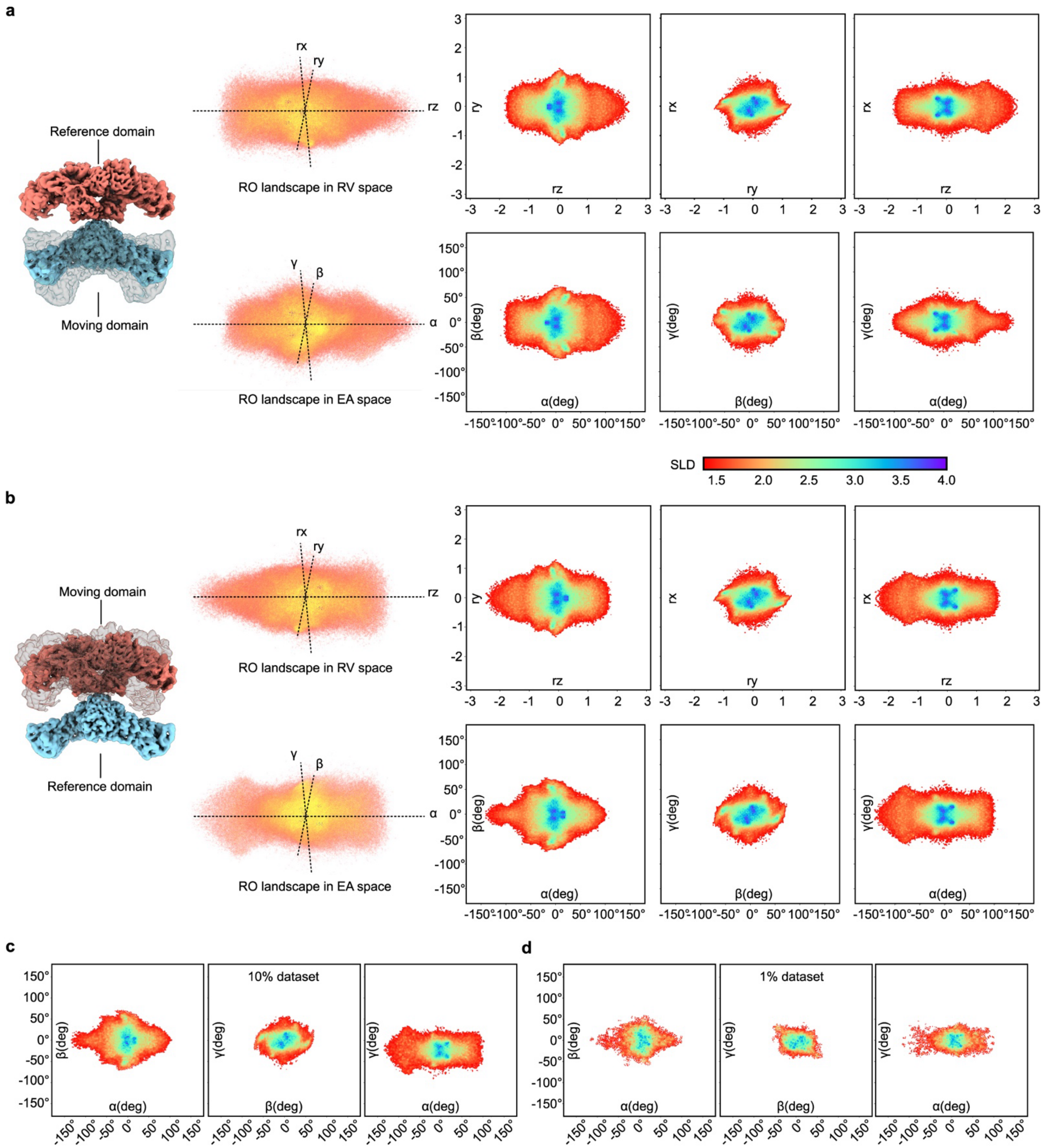

Supplementary Figure 2 Effect of exchanging reference and moving domains

a and b, Moving and reference domains are swapped in the composite map of hFASN, producing an inverted landscape in RV space and the corresponding re-parameterized landscape in EA space, without changing the intrinsic landscape structure. **c and d**, RO landscapes from random 10% and 1% subsets of the FASN dataset.

bioRxiv preprint doi: https://doi.org/10.64898/2026.07.04.736454; this version posted July 4, 2026. The copyright holder for this preprint (which was not certified by peer review) is the author/funder, who has granted bioRxiv a license to display the preprint in perpetuity. It is made available under a CC-BY 4.0 International license.

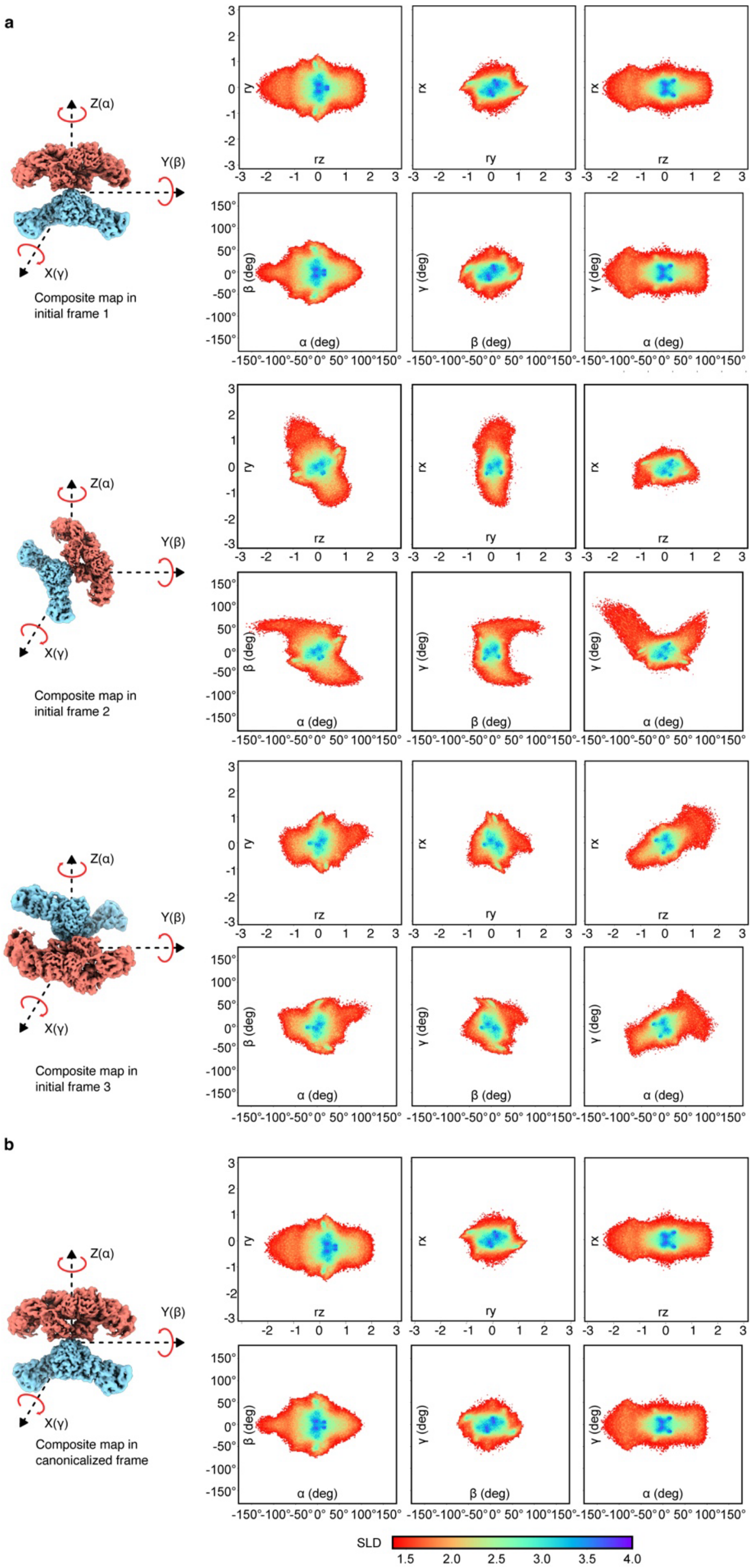

Supplementary Figure 3 Effect of reorienting the reference composite map

a, RO landscapes of the same FASN dataset displayed using three different initial composite-map coordinate frames. Changing the initial frame rotates the RO cloud linearly in rotation-vector (RV) space and produces nonlinear changes in Euler-angle displays. **b,** After canonicalization, the corresponding RO landscapes converge to the same canonical representation.

bioRxiv preprint doi: https://doi.org/10.64898/2026.07.04.736454; this version posted July 4, 2026. The copyright holder for this preprint (which was not certified by peer review) is the author/funder, who has granted bioRxiv a license to display the preprint in perpetuity. It is made available under a CC-BY 4.0 International license.

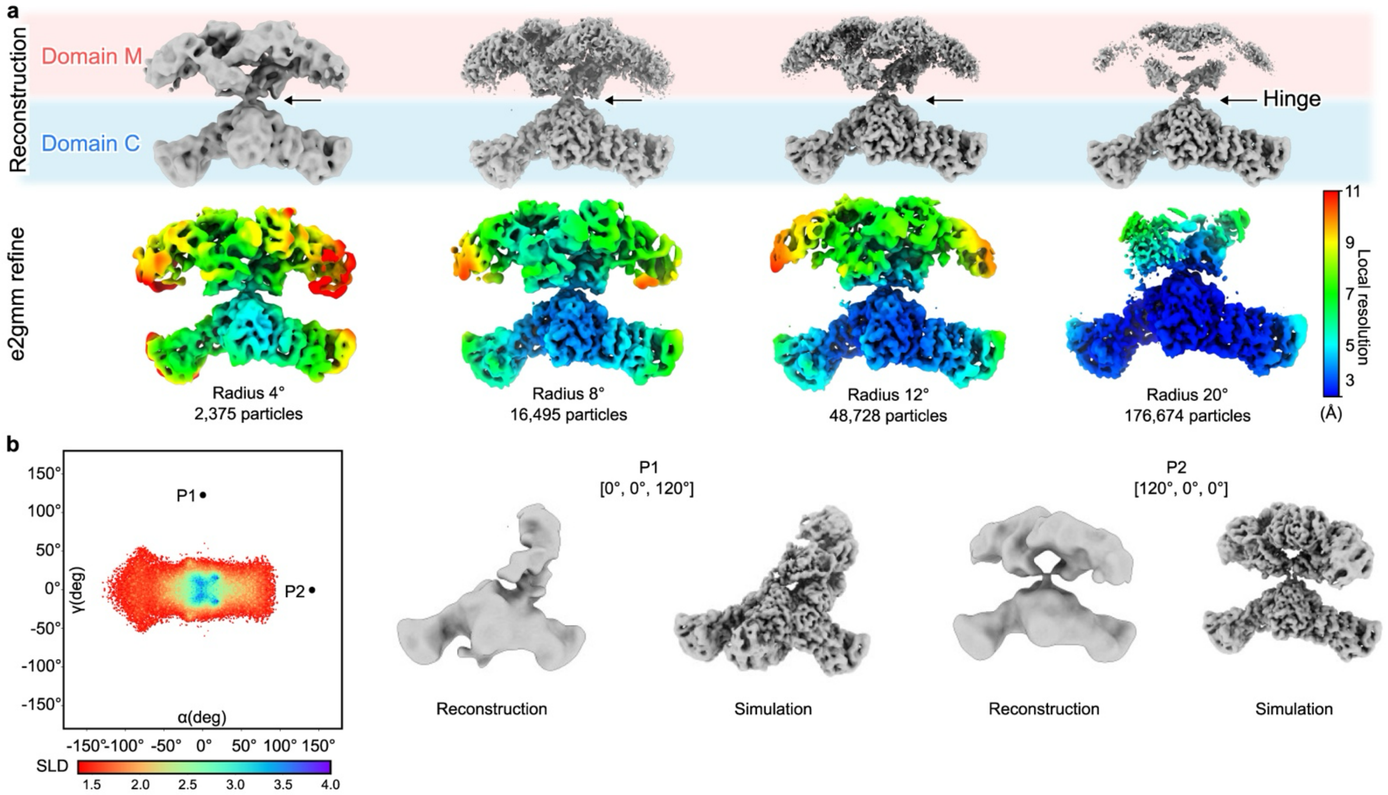

Supplementary Figure 4 Practical considerations for RO landscape-guided reconstruction.
a, Reconstructions from particles selected around the same FASN RO coordinate using increasing geodesic radii. Larger radii increase particle number but reduce RO homogeneity, leading to progressive blurring of the moving domain. Bottom row, e2GMM-refined maps colored by local resolution. **b,** Reconstructions from low-SLD coordinates compared with corresponding simulations.

bioRxiv preprint doi: https://doi.org/10.64898/2026.07.04.736454; this version posted July 4, 2026. The copyright holder for this preprint (which was not certified by peer review) is the author/funder, who has granted bioRxiv a license to display the preprint in perpetuity. It is made available under a CC-BY 4.0 International license.

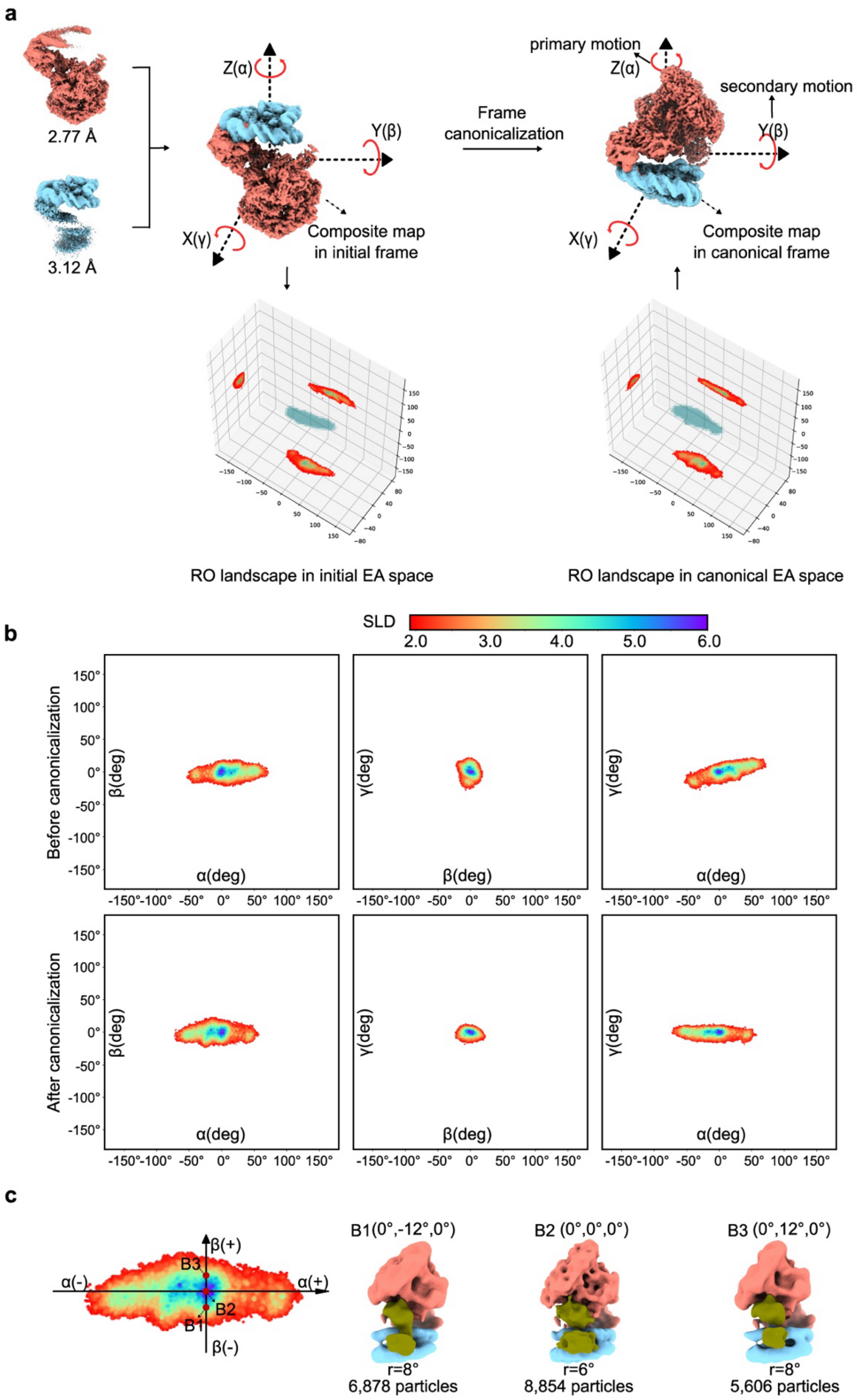

Supplementary Figure 5 Motion-aligned frame canonicalization of the INO80-hexasome RO landscape. **a,** INO80 and hexasome focused-refinement maps were assembled into a composite map and reoriented from the initial frame to the canonical frame. Corresponding RO landscapes are shown in fixed-axis Z–Y–X Euler-angle space. **b,** Two-dimensional RO projections before and after canonicalization. **c,** Reconstructions from particles selected along the secondary β coordinate at B1, B2 and B3.

bioRxiv preprint doi: https://doi.org/10.64898/2026.07.04.736454; this version posted July 4, 2026. The copyright holder for this preprint (which was not certified by peer review) is the author/funder, who has granted bioRxiv a license to display the preprint in perpetuity. It is made available under a CC-BY 4.0 International license.

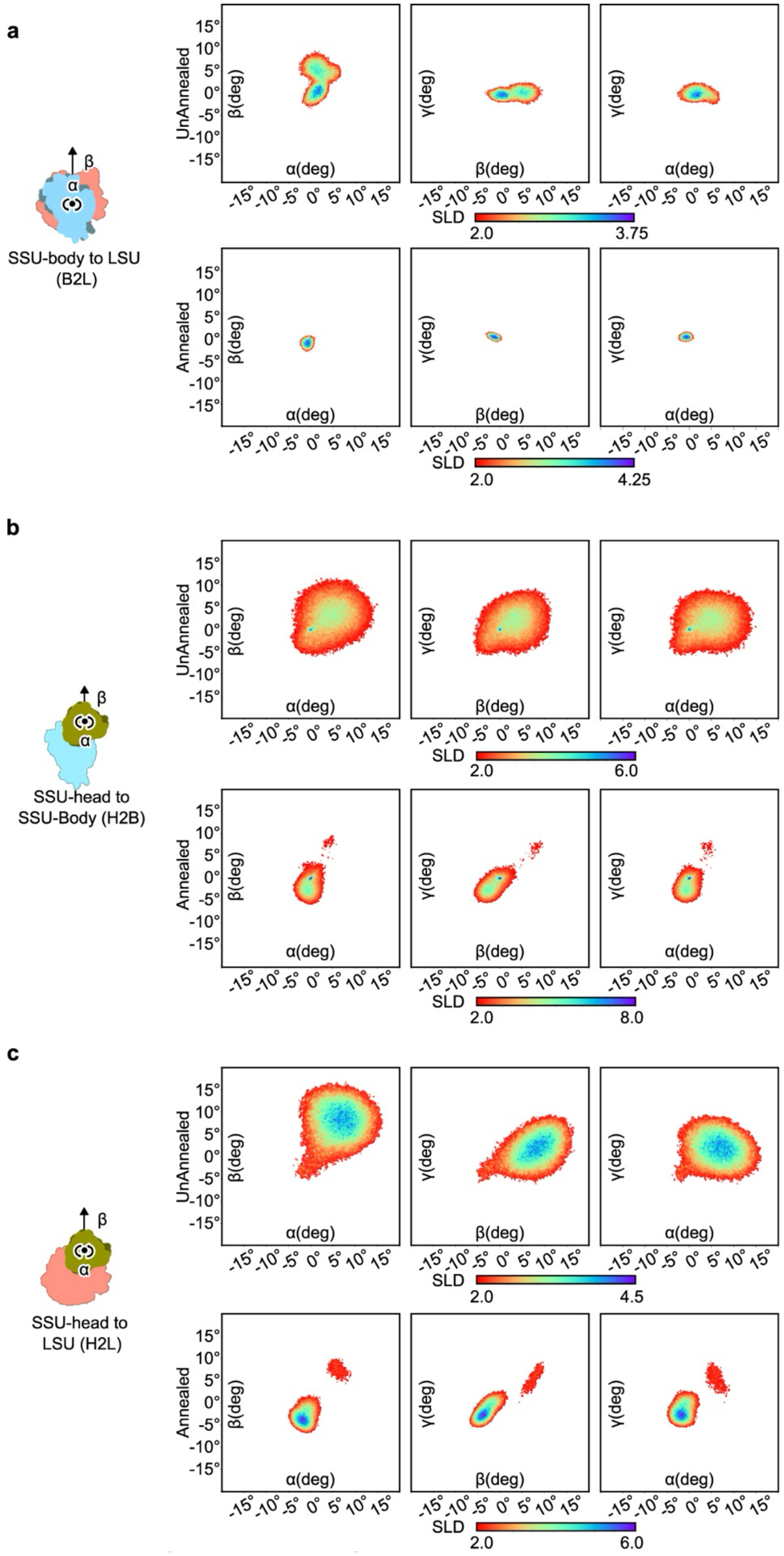

Supplementary Fig. 6 | RO landscapes of ribosomal domain motions without and after temperature annealing.

a–c, Two-dimensional projections of ribosome RO landscapes for the unannealed and annealed datasets. Three pairwise domain motions are SSU-body to LSU (B2L, **a**), SSU-head to SSU-body (H2B **b,**) and SSU-head to LSU (H2L, **c**).

bioRxiv preprint doi: https://doi.org/10.64898/2026.07.04.736454; this version posted July 4, 2026. The copyright holder for this preprint (which was not certified by peer review) is the author/funder, who has granted bioRxiv a license to display the preprint in perpetuity. It is made available under a CC-BY 4.0 International license.

a

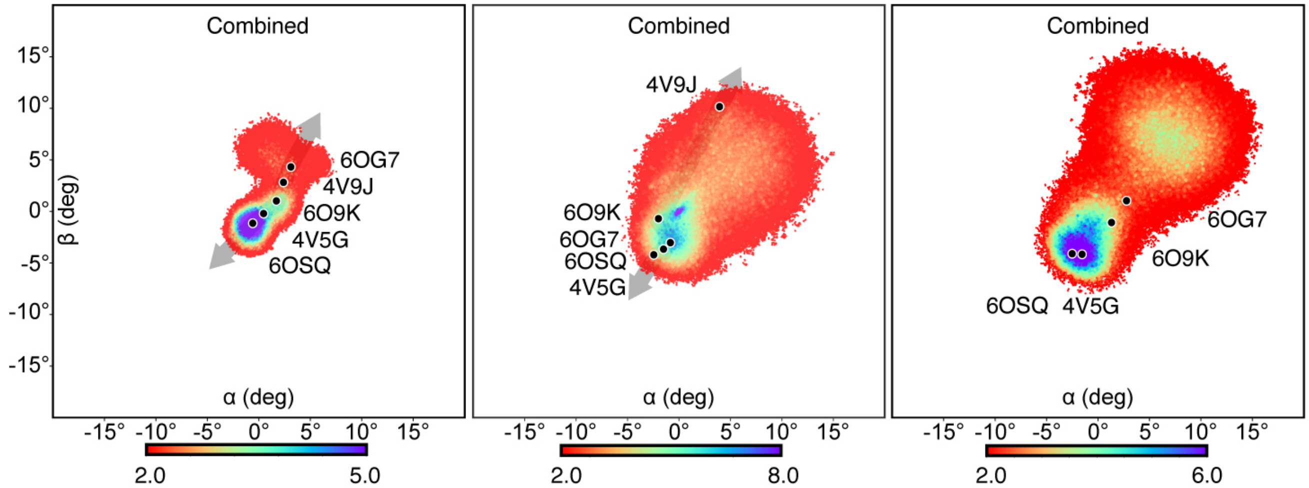

b

PDB	State	B2L			H2B			H2L		
6O9K	initiation	1.597	0.817	0.818	-0.707	-2.010	-1.939	0.917	-1.247	-1.064
5AFI	Decoding	0.438	-0.005	-0.244	-2.421	-3.741	-3.167	-1.983	-3.770	-3.383
8BF7	Accommodation	0.947	0.910	-0.140	-2.481	-3.658	-2.598	-1.492	-2.791	-2.675
6OSQ	RF1 accommodated	-0.753	-0.949	-0.078	-1.483	-2.752	-2.696	-2.282	-3.665	-2.813
6OGF	RF2 rearrangement	3.090	3.807	0.585	-0.818	-2.874	-2.656	2.439	0.789	-1.909
6GWT	RF3-mediated	-0.520	-0.288	-0.421	-1.665	-2.794	-1.971	-2.195	-3.065	-2.418
7SSN	PRE-hibird	7.622	4.493	-2.042	0.567	-0.638	0.067	8.178	3.869	-1.891
7SSD	Mid-translocate	2.221	0.893	-0.217	4.399	8.842	9.018	6.485	10.066	8.475
7SS9	Late-translocate	-0.478	-0.954	0.021	5.124	10.557	8.645	4.790	9.541	8.728
7ST2	Fully-translocate	-1.306	-1.347	0.012	-0.967	-2.496	-2.988	-2.345	-3.773	-3.035

Supplementary Fig. 7 | Mapping known ribosome states onto the combined RO landscapes.
a, Published *E. coli* 70S ribosome structures were mapped onto the combined RO landscapes for B2L, H2B, and H2L motions. Black dots mark the mapped coordinates of representative functional states. **b,** Table of mapped α, β, and γ coordinates for each PDB structure in the three RO landscapes.

bioRxiv preprint doi: https://doi.org/10.64898/2026.07.04.736454; this version posted July 4, 2026. The copyright holder for this preprint (which was not certified by peer review) is the author/funder, who has granted bioRxiv a license to display the preprint in perpetuity. It is made available under a CC-BY 4.0 International license.

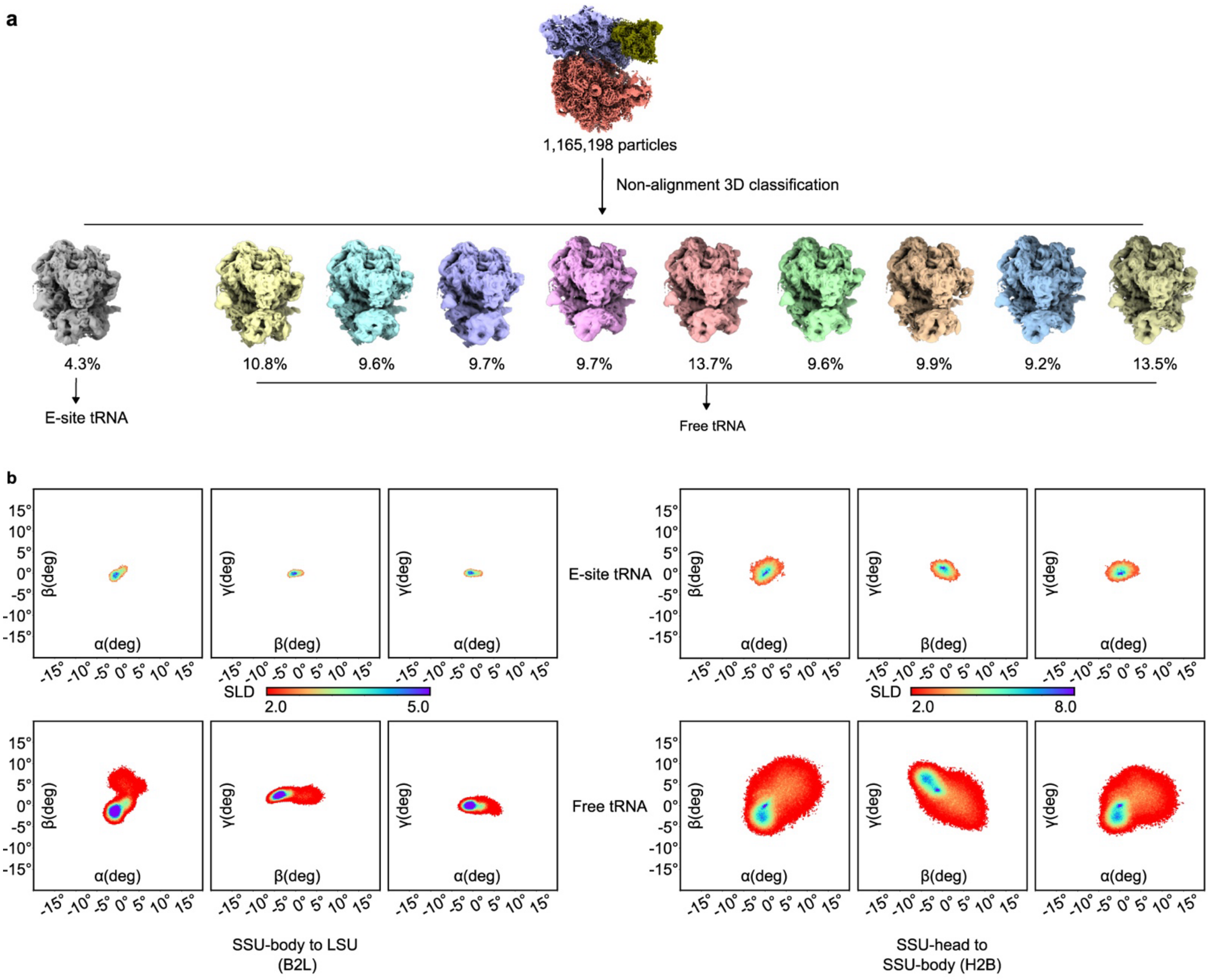

Li and Choi, et al., Supplementary Figuer 8

Supplementary Fig. 8 | E-site tRNA binding is associated with a restricted ribosome RO landscape. **a,** The combined ribosome dataset was subjected to non-alignment 3D classification. One minor class (4.3%) showed clear density for an E-site tRNA, whereas the remaining classes lacked detectable tRNA density and were grouped as the tRNA-free population. **b,** Two-dimensional projections of the RO landscapes of B2L and H2B for the E-site tRNA-bound and tRNA-free populations.

bioRxiv preprint doi: https://doi.org/10.64898/2026.07.04.736454; this version posted July 4, 2026. The copyright holder for this preprint (which was not certified by peer review) is the author/funder, who has granted bioRxiv a license to display the preprint in perpetuity. It is made available under a CC-BY 4.0 International license.

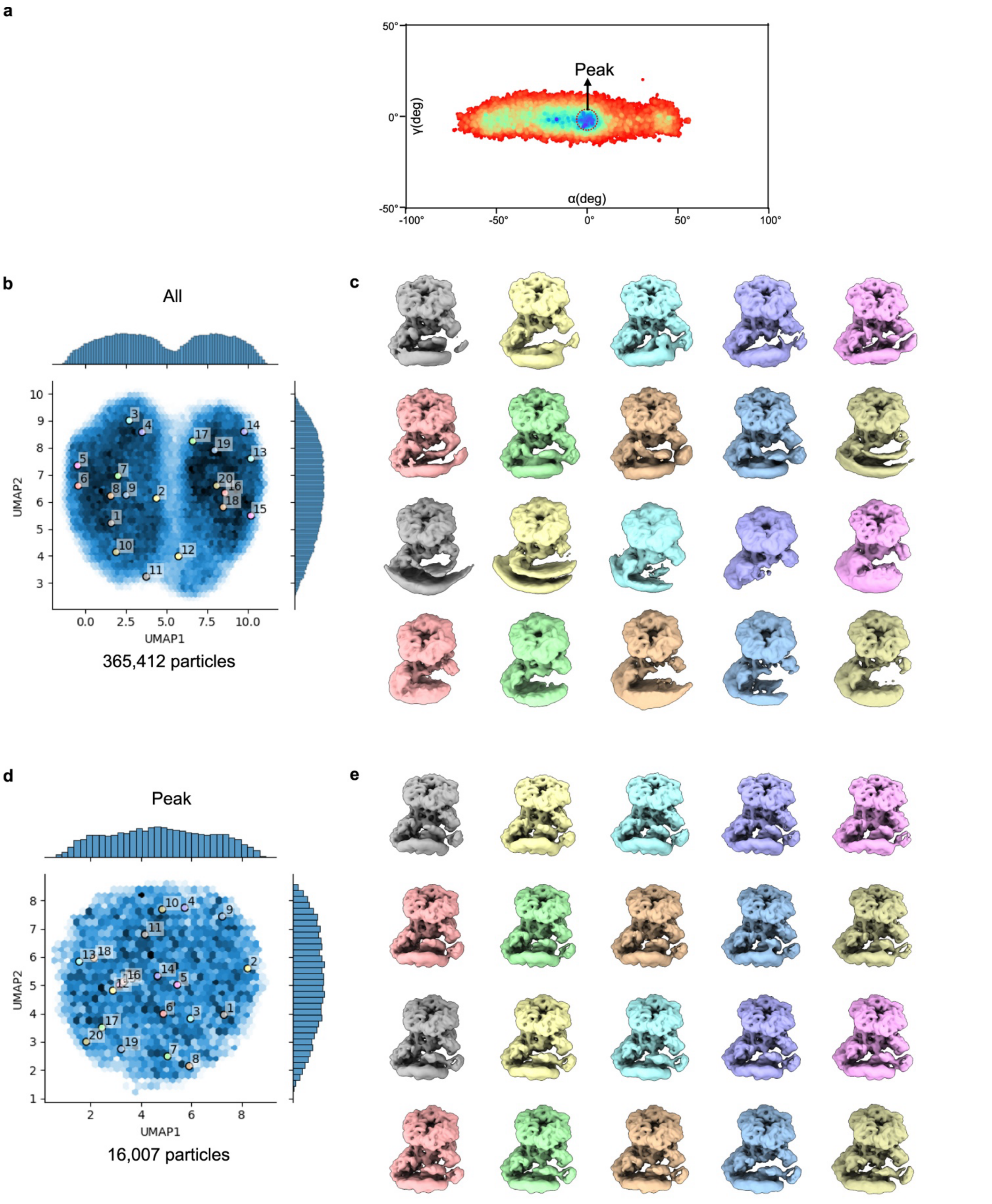

Supplementary Fig. 9 | CryoDRGN analysis of the full INO80-hexasome dataset and of a cryoROLE-selected local subset.

a, Peak (0°,0°,0°) in the canonicalized RO landscape. **b** and **c,** UMAP embedding and k-means (k=20) reconstructions from cryoDRGN analysis of the full dataset (365,412 particles). **d** and **e,** UMAP embedding and k-means ((k=20)) reconstructions from cryoDRGN analysis of particles selected within an 8° geodesic radius around Peak (16,007 particles). Both analyses were performed with the same default cryoDRGN parameters.
